# Flavanomarein inhibits high glucose-stimulated epithelial-mesenchymal transition in HK-2 cells via targeting spleen tyrosine kinase

**DOI:** 10.1038/s41598-019-57360-4

**Published:** 2020-01-16

**Authors:** Nan-nan Zhang, Jin-sen Kang, Shuai-Shuai Liu, Si-Meng Gu, Zhi-peng Song, Feng-xiang Li, Li-feng Wang, Lan Yao, Tian Li, Lin-lin Li, Ye Wang, Xue-jun Li, Xin-min Mao

**Affiliations:** 1 State Key Laboratory of Pathogenesis, Prevention, Treatment of Central Asian High Incidence Diseases, Xinjiang Medical University, Urumqi, Xinjiang 830011 China; 2 Department of Pharmacology, Pharmacy College, Xinjiang Medical University, Urumqi, Xinjiang 830011 China; 3 Department of Pharmacology, School of Basic Medical Sciences, Peking University, Beijing, 100191 China; 4 Department of Physiology, Preclinical School, Xinjiang Medical University, Urumqi, Xinjiang 830011 China; 5 College of Traditional Chinese Medicine, Xinjiang Medical University, Urumqi, Xinjiang 830011 China; 6 Department of Histology and Embryology, Preclinical College, XinJiang Medical University, Urumqi, Xinjiang 830011 China

**Keywords:** Biochemistry, Target identification, Renal fibrosis

## Abstract

Flavanomarein (FM) is a major natural compound of *Coreopsis tinctoria Nutt* with protective effects against diabetic nephropathy (DN). In this study, we investigated the effects of FM on epithelial-mesenchymal transition (EMT) in high glucose (HG)-stimulated human proximal tubular epithelial cells (HK-2) and the underlying mechanisms, including both direct targets and downstream signal-related proteins. The influence of FM on EMT marker proteins was evaluated via western blot. Potential target proteins of FM were searched using Discovery Studio 2017 R2. Gene Ontology (GO) analysis was conducted to enrich the proteins within the protein-protein interaction (PPI) network for biological processes. Specific binding of FM to target proteins was examined via molecular dynamics and surface plasmon resonance analyses (SPR). FM promoted the proliferation of HK-2 cells stimulated with HG and inhibited EMT through the Syk/TGF-β1/Smad signaling pathway. Spleen tyrosine kinase (Syk) was predicted to be the most likely directly interacting protein with FM. Combined therapy with a Syk inhibitor and FM presents significant potential as an effective novel therapeutic strategy for DN.

## Introduction

DN is one of the major complications of diabetes^1^ and a predominant cause of end-stage renal failure^2^. The pathological features of DN include accumulation of extracellular matrix and interstitial fibrosis^3^. High glucose level is an independent risk factor for DN^4^ and elevated glucose can promote EMT through regulation of different pathways. HG conditions are reported to stimulate the production of a number of critical fibrogenic cytokines and the process of the EMT^5,6^. Accelerated renal fibrosis is one of the mechanisms underlying DN. EMT plays an important role in renal fibrosis, in particular, renal tubulointerstitial fibrosis^7–9^. More than one-third of the renal interstitial myofibroblasts originate from renal tubular epithelial cells^10^. EMT describes a complex set of epithelial cell phenotypic transitions in which epithelial cells lose their cell-cell-basement membrane contacts and their structures become fusiform, similar to mesenchymal myofibroblasts^11^. E-cadherin, α-SMA and Vimentin proteins are the most commonly used markers to indicate the development of EMT. FN and type I collagen (collagen I) have been identified as the main extracellular matrix components. TGF-β1 is an important growth factor that induces EMT and promotes tubulointerstitial fibrosis^12–15^. Here, we explored the antifibrotic effect of FM on HG-induced EMT in HK-2 cells and the underlying mechanisms, with a view to developing novel therapeutic drug targets for DN.

Coreopsis tinctoria Nutt is generally used as a health tea owing to its anti-hyperlipidemic, anti-inflammatory and glycemia regulation activities^16,17^. The main active components of the extracts include phenolic acids (chlorogenic acid, caffeic acid) and different flavonoids (flavanomarein, marein, and okanin aurone)^18^. Flavanomarein and marein contents are generally the highest in Coreopsis tinctoria Nutt extracts, and may play a key role in quality assessment^19^. Previous experiments by our group showed that FM was absorbed into the blood as a prototype^20^.

In the current study, we assessed whether FM exerts inhibitory activity against HG-induced EMT in HK-2 cells and further explored its direct downstream targets and underlying mechanisms of action.

## Results

### FM promotes HK-2 cell proliferation

**Figure 1:**
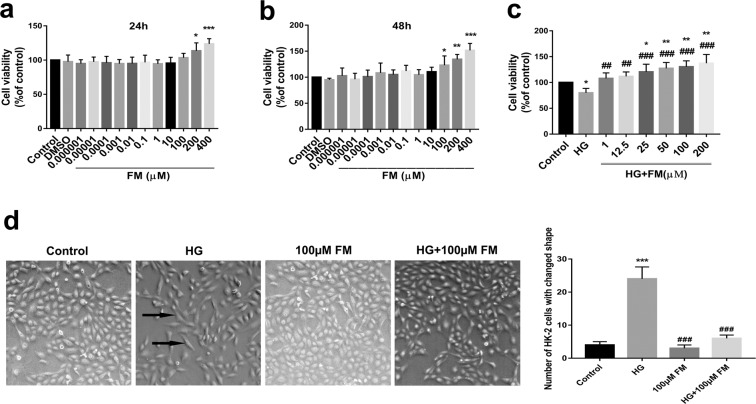
Effects of FM on viability of HK-2 cells (MTS assay) and cell morphology. (**a**,**b**) Effects of FM on viability of HK-2 cells. (**c**) Effects of FM on viability of HK-2 cells stimulated with HG for 48 h. (**d**) Effects of FM on morphology of HK-2 cells stimulated with HG for 48 h (50×). Black arrows indicate cells that have undergone EMT. Data are presented as means ± SD, *n* = 3 (three separate experiments). ^*^P < 0.05, ^**^P < 0.01, ^***^P < 0.001 compared with control and ^##^*P* < 0.01, ^###^*P* < 0.001, compared with HG (HG, high glucose; FM, flavanomarein).

As shown in Fig. 1(a,b), data from the MTS assay revealed that FM significantly enhances cell viability at 24 h (200 or 400 µM) and 48 h (100, 200 or 400 µM), clearly supporting FM-mediated induction of HK-2 proliferation. As shown in Fig. 1c, HG-treated HK-2 cells displayed significantly decreased cell viability at 48 h (~80% that of the control group). Treatment with FM at concentrations of 25, 50, 100, and 200 µM induced a marked dose-dependent increase in cell viability, compared with the control group. Notably, cell viability was increased in the FM treatment groups (1, 12.5, 25, 50, 100, and 200 µM), compared with the HG group, in a dose-dependent manner. HG-induced HK-2 cells showed significant changes in morphology from cobblestone-like to long spindle-shaped cells (marked with black arrows), characteristic of EMT^21^. In the presence of 100 µM FM, the degree of EMT was reduced (Fig. 1d).

### FM inhibits the expression of α-SMA, FN and Vimentin while enhancing E-cadherin in HG-treated HK-2 cells

**Figure 2:**
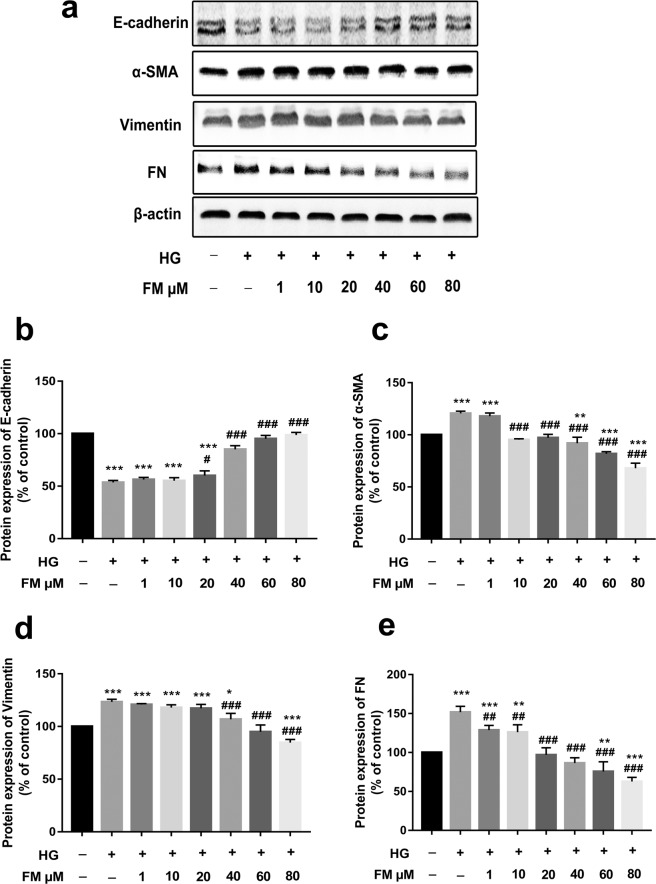
FM inhibits expression of α-SMA, FN and Vimentin while enhancing that of E-cadherin in HG-treated HK-2 cells. (**a**) Western blot analysis of E-cadherin, α-SMA, Vimentin and FN. (**b**–**e**) Statistical analysis of western blot data for E-cadherin, α-SMA, Vimentin, and FN. Data are presented as means ± SD, n = 3 (three separate experiments). ^*^P < 0.05, ^**^P < 0.01, ^***^P < 0.001, compared with control, and ^#^*P* < 0.05, ^##^*P* < 0.01, ^###^*P* < 0.001, compared with HG (HG, high glucose; FM, flavanomarein).

To confirm whether FM regulates the EMT process, the expression of EMT marker proteins, such as E-cadherin, α-SMA, Vimentin, and FN, was examined via western blot. As shown in Fig. 2, compared with the control group, E-cadherin expression was significantly decreased, and conversely, levels of the three other proteins were significantly increased in the HG group. Relative to the HG group, FM (40, 60, and 80 µM) induced a considerable concentration-dependent increase in the E-cadherin level. Moreover, FM treatment at concentrations of 1, 10, 20, 40, 60, and 80 µM led to a significant dose-dependent decrease in α-SMA, Vimentin and FN levels, clearly indicating an anti-EMT effect on HG-stimulated HK-2 cells.

### Prediction of potential targets of FM via a computational reverse docking approach

**Figure 3:**
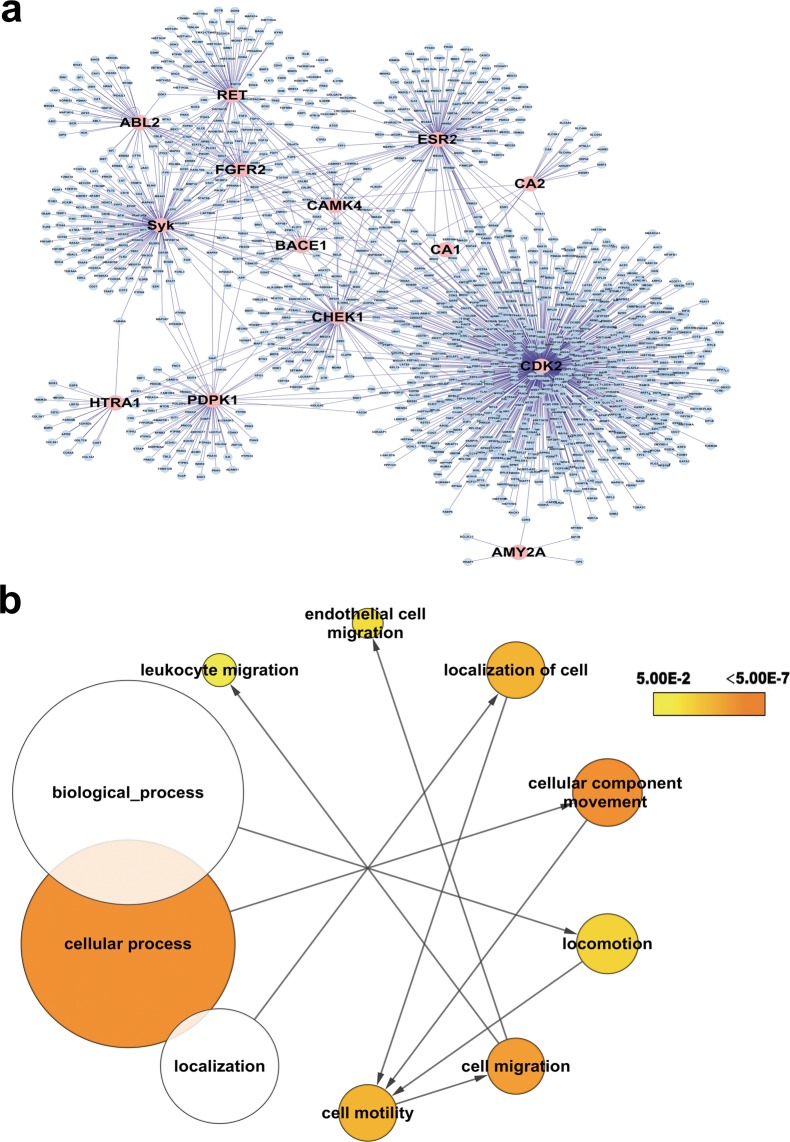
Identification of key proteins contributing to the effects of FM on cell migration. (**a**) Protein-protein interaction (PPI) network derived from reverse target screening. The PPI network comprises 1254 nodes and 1463 edges, with pink nodes representing the 15 human seed proteins. (**b**) Gene Ontology (GO) analysis was conducted on the PPI network to enrich nodes based on their “biological processes” annotation. The structure of cell motility-relevant GO and root terms are depicted. The color of the GO term represents the p-value and size signifies the number of enriched proteins.

In total, 43 proteins were identified, including 15 human proteins. In order to establish the key proteins contributing to the effects of FM on cell migration, we constructed a PPI network of the 15 human proteins based on the results of reverse target screening incorporating 1254 nodes and 1463 interactions (Fig. 3a). Subsequently, GO analysis was performed to categorize all proteins within the PPI network based on the “biological process” they are involved in (Fig. 3b). We focused on the cell motility-relevant GO terms “cell migration” and “cell motility” and their mother terms. Intriguingly, RET and Syk appeared in all the cell migration and motility-relevant GO terms (Table 1). Notably, Syk mediates upregulation of high glucose-induced TGF-β1 in HK-2 cells^22^ and is involved in inflammation. TGF-β1 is a classical regulatory cytokine and an important inducer of renal tubular epithelial cell transdifferentiation. The nodes and interactions of RET are lower than those of Syk, as shown in Fig. 3a. We hypothesize that Syk plays a critical role in promoting the activity of FM.

**Table 1:** Cell migration-relevant GO terms.

Go-ID	Description	p-value	Cluster freq	Seed freq	Seed Gene
16477	Cell migration	2.0307E-07	49/1146 4.2%	3/49	RET Syk ESR2
48870	Cell motility	2.4724E-06	50/1146 4.3%	3/50	RET Syk ESR3
40011	Locomotion	1.2836E-04	58/1146 5.0%	3/58	RET Syk ESR4
6928	Cellular component movement	7.7116E-10	78/1146 6.8%	1/26	RET Syk ESR5
51674	Localization of cell	2.4724E-06	50/1146 4.3%	3/50	RET Syk ESR6

### Syk is a direct protein target of FM

**Figure 4:**
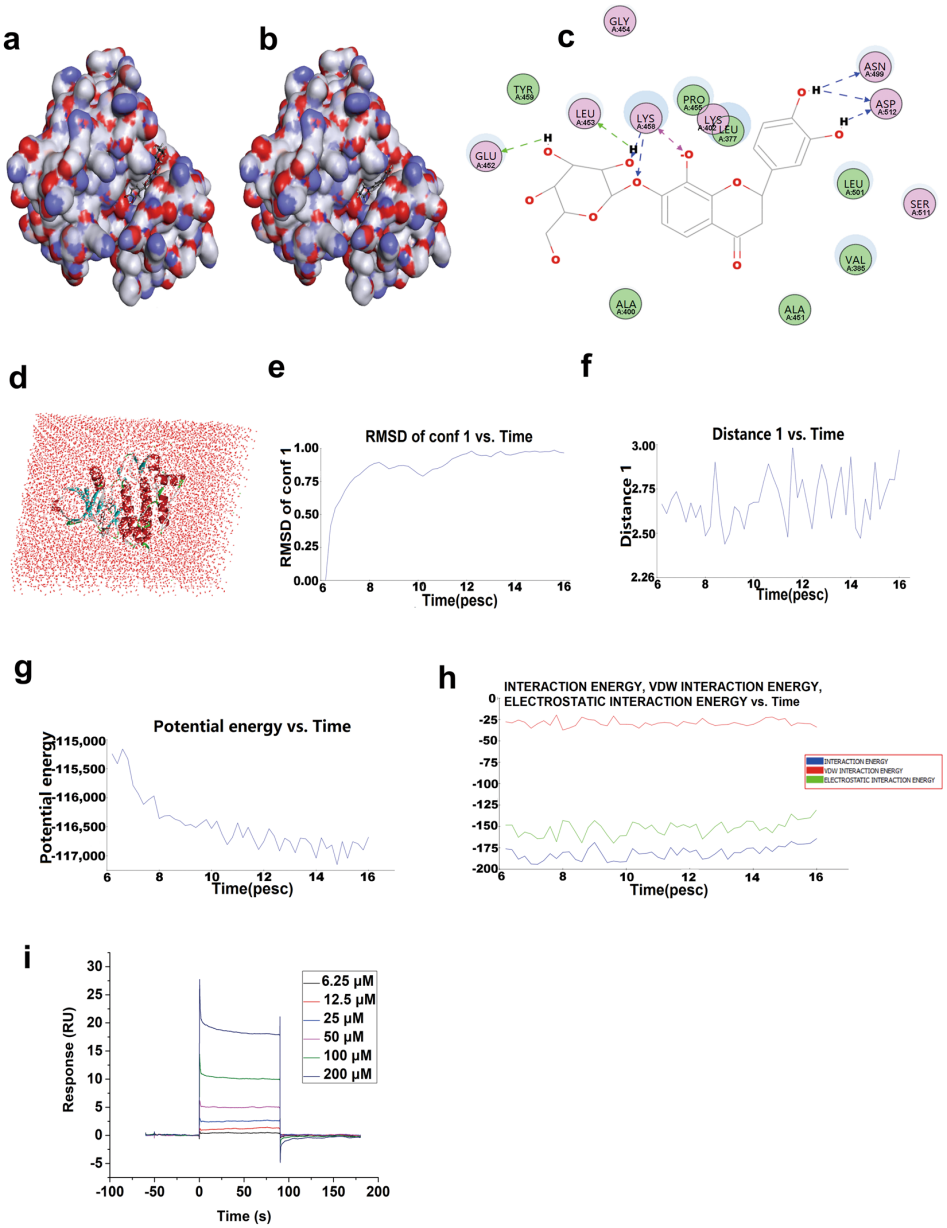
Protein-ligand interactions, molecular dynamics and binding affinity analysis of Syk and FM. (**a**) Interaction models of Syk and FM in the optimal docking pose. The -CDOCKER_INTERACTION_ENERGY score was −75.1069 kcal/mol. (**b**) Interaction models of Syk and ligand LASW836 in the optimal docking pose. The -CDOCKER_INTERACTION_ENERGY score was −57.4404 kcal/mol. (**c**) Detailed interaction modes of Syk and FM in the optimal docking pose. (**d**) Model of the Syk-FM complex in solvent. (**e**) Drug positional RMSD. (**f**) Distance between O of FM and HN in the amino residue of Lys458 in Syk. (**g**) Potential energy of the amino residue group between Syk and FM. (**h**) Interaction energy of the amino residue group between Syk and FM evaluated using molecular dynamics. (**i**) Real-time binding affinity measurements of FM using Biacore T200. Representative sensorgrams obtained from injection of different concentrations of FM (6.25, 12.5, 25, 50, 100, and 200 μM; curves from bottom to top) over the immobilized Syk surface on the CM5 chip. Note: FM is displayed in the stick representation while residues of Syk are presented as balls. Water is depicted in pink.

To investigate the interactions of Syk with FM, specific binding between the two molecules was examined via molecular dynamics and SPR analyses. Data from *in silico* drug target docking modeling indicated that FM directly enters the binding pocket of Syk (Fig. 4a–d) with −75.1069 kcal/mol in the optimal binding pose, showing better binding energy than the endogenous ligand LASW836 (−57.4404 kcal/mol). As shown in Fig. 4c, Lys458, Asn499, Asp512, Leu453 and Glu452 play decisive roles in hydrogen bond formation, in particular, Lys458, which contributes to stabilizing the complex of Syk and FM. A model of the complex of Syk bound to FM in solvent is presented in Fig. 4d. The RMSD reference of FM, plotted in Fig. 4e, showed that interactions of the receptor-ligand complex reach the equilibrium state after 12 pescs. A similar situation was observed in the analysis of interactions between O of FM and HN in the amino residue of Lys458 in Syk (Fig. 4f–h), suggesting that these two residues of the catalytic site stabilize the interactions between FM and Syk. A hydrogen bond heat map of the Syk-FM complex is presented in Supplemental Fig. 1. The ordinate represents all possible hydrogen bonds in the protein and the vertical coordinates are the steps in the simulation, indicating activation of hydrogen bonds in each step. We additionally investigated the binding affinity of FM for Syk based on SPR. The response unit (RU) values increased significantly with incremental FM doses from 6.25 to 200 μM (Fig. 4i), indicating that FM directly binds Syk in a concentration-dependent manner. The equilibrium dissociation constant of FM binding to immobilized Syk on a CM5 chip (K_D_ = kd/ka) was 3.064 × 10^−5^ M, supporting the theory that Syk is a direct target of FM.

### A Syk inhibitor inhibits α-SMA, FN, and Vimentin and increases E-cadherin expression in HG-treated HK-2 cells

**Figure 5:**
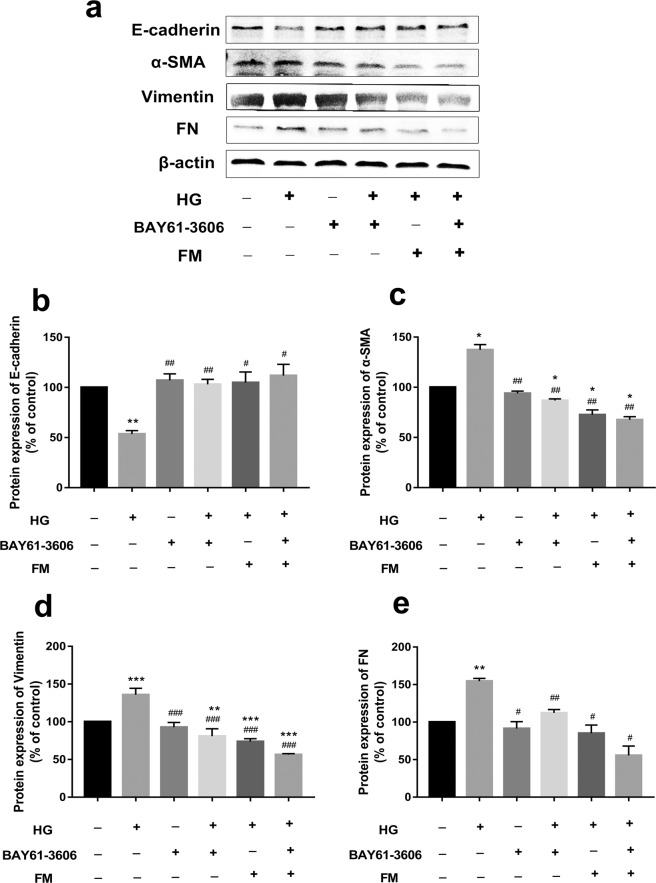
The Syk inhibitor, BAY61-3606, inhibits expression of α-SMA, Vimentin, and FN and enhances E-cadherin expression. (**a**) Western blot analysis of E-cadherin, α-SMA, Vimentin and FN. (**b**–**e**) Statistical analysis of western blots for E-cadherin, α-SMA, Vimentin, and FN. Data are presented as means ± SD, n = 3 (three separate experiments). ^*^P < 0.05, ^**^P < 0.01, ^***^P < 0.001, compared with control and ^#^*P* < 0.05, ^##^*P* < 0.01, ^###^*P* < 0.001, compared with HG (Note: 80 µM FM; 1 µM BAY61-3606;HG, high glucose; FM, flavanomarein).

To validate whether Syk is a direct target of FM, HG-exposed HK-2 cells were treated with BAY61-3606, a potent, ATP-competitive, and highly selective inhibitor of Syk tyrosine kinase with no suppressive effects on Lyn, Btk, Fyn, Itk and Src. Protein expression of E-cadherin, Vimentin, α-SMA, and FN in a diabetic kidney model was detected via western blot, as shown in Fig. 5. Compared with the control group, the HG group showed a significant decrease in E-cadherin, and conversely, a significant increase in α-SMA, Vimentin, and FN levels. Relative to the HG group, E-cadherin expression was markedly increased in the group co-treated with FM (80 µM) and the Syk inhibitor, BAY61-3606 (1 µM). The FM + BAY61-3606 treatment group displayed the highest increase in E-cadherin overall. Moreover, FM, BAY61-3606, and FM + BAY61-3606 treatment caused a marked decrease in the levels of α-SMA, Vimentin, and FN, compared with the HG group. Our results suggest that Syk is implicated in the anti-EMT effect of FM.

### The Syk inhibitor ameliorates renal EMT through suppression of the Syk/TGF-β1/Smad signaling pathway

**Figure 6:**
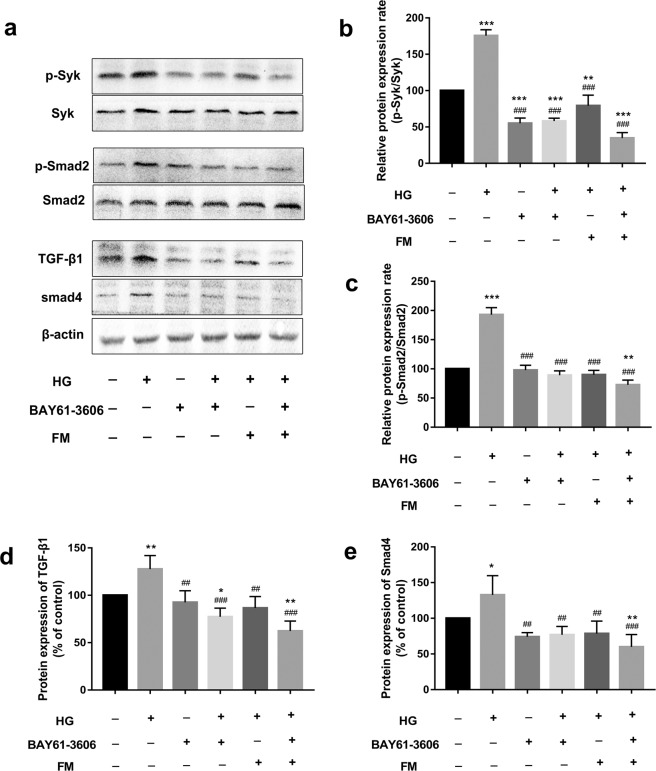
The Syk inhibitor ameliorates EMT through suppression of the Syk/TGF-β1/Smad signaling pathway. (**a**) Western blot analysis of p-Syk/Syk, TGF-β1, Smad4, p-Smad2/Smad2 expression in HK-2 cells. (**b**–**e**) Statistical analysis of western blots for p-Syk/Syk, TGF-β1, Smad4, p-Smad2/Smad2 in HK-2 cells. Data are presented as means ± SD, n = 3 (three separate experiments). ^*^P < 0.05, ^**^P < 0.01, compared with control and ^#^*P* < 0.05, ^##^*P* < 0.01, ^###^*P* < 0.001, compared with HG (Note: 80 µM FM; 1 µM BAY61-3606; HG,high glucose;FM,flavanomarein).

To elucidate the mechanism underlying the anti-EMT effects of FM, components of the Syk/TGF-β1/Smad signaling pathway, considered to play a key role in EMT, were investigated via western blot. As shown in Fig. 6, p-Syk levels in HK-2 cells were significantly increased, followed by increase in TGF-β1, p-Smad2, and Smad4 levels. p-Syk, TGF-β1, p-Smad2, and Smad4 expression was markedly decreased upon treatment with 80 µM FM and/or 1 µM BAY61-3606, supporting the theory that the anti-EMT effects of FM are associated with the Syk/TGF-β1/Smad signaling pathway.

### Syk-siRNA ameliorates renal EMT by suppressing the Syk/TGF-β1/Smad signaling pathway

**Figure 7:**
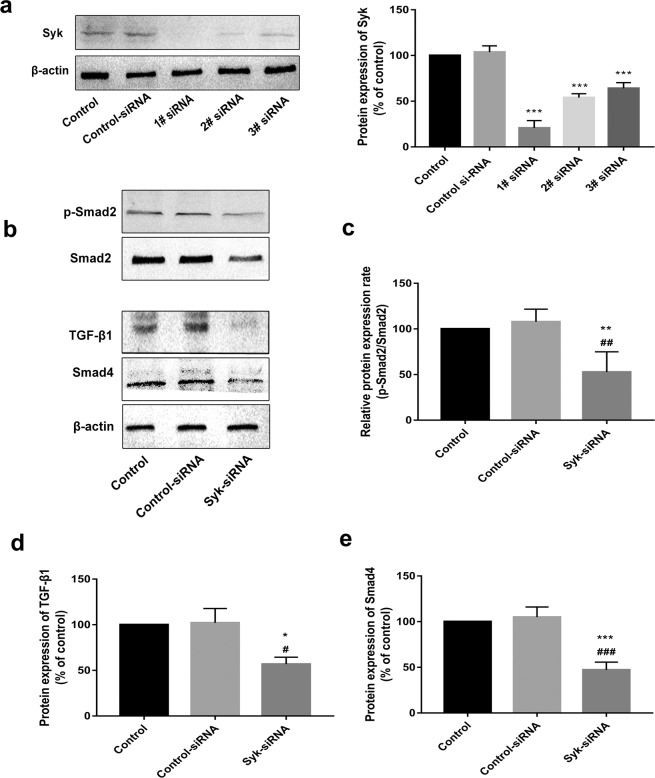
Syk siRNA ameliorates renal EMT by suppressing the Syk/TGF-β1/Smad signaling pathway. (**a**) Gene silencing effect of Syk siRNA. (**b**) Western blot analysis of TGF-β1, Smad, p-Smad2 and Smad4 in HK-2 cells. (**c**–**f**) Statistical analysis of western blot data on TGF-β1, p-Smad2 and Smad4 in HK-2 cells. Data are presented as means ± SD, n = 3 (three separate experiments). ^*^P < 0.05, ^**^P < 0.01, ^***^P < 0.001, compared with control, and ^#^*P* < 0.05, ^###^*P* < 0.001, compared with control siRNA (control siRNA, negative scramble control siRNA).

To elucidate the mechanism underlying the anti-EMT effects of FM, Syk -siRNA was transfected into HK-2 cells. As shown in Fig. 7a, the gene silencing effect of 1# siRNA was greatest, which was therefore selected for follow-up experiments. Compared with levels in the control group, TGF-β1, p-Smad2, and Smad4 expression in the Syk siRNA group was significantly decreased (Fig. 7b–f), further indicating that the inhibitory effects of FM on EMT are associated with Syk /TGF-β1/Smad signaling.

## Discussion

Coreopsis tinctoria Nutt, also known as Kunlun snow chrysanthemum, is predominantly distributed in the South Xinjiang region of China^23^. Earlier studies by our group confirmed that the ethyl acetate extract exerts a renal protective effect in high-glucose-fat diet and streptozotocin-induced diabetic rats^24^. Its component eriodictyol 7-O-β-D glucopyranoside could ameliorate lipid disorders via suppressing lipogenesis and protecting mitochondrial function^25^. In streptozotocin-induced glucose-intolerant rats, the flavonoid-rich fraction exerted a cytoprotective effect on tBHP and cytokine-induced cell injury in MIN6 cells^26^. Extracts of Coreopsis tinctoria Nutt. flower exert antidiabetic effects via inhibiting α-glucosidase activity^27^. As the main active component of Coreopsis tinctoria Nutt, FM has attracted significant research attention. However, the direct target proteins of FM and downstream signaling network are yet to be established. In the present study, 43 potential direct target proteins (including 15 human-related proteins) of FM were predicted using a ligand–protein inverse-docking algorithm, and a PPI network constructed based on the data obtained with the 15 proteins. GO analysis was conducted to enrich the proteins of the PPI network for biological processes. Based on the findings, we hypothesized that Syk plays a critical role in facilitating the effect of FM. Examination of specific interactions via molecular dynamics and SPR analyses confirmed that FM binds Syk in a dose-dependent manner.

Syk, a non-receptor tyrosine kinase, is a member of the protein tyrosine kinase family^28^ characterized by intracellular signal cascades, such as activated Fc and B cell receptors^29^. Syk is critical for mediating Fc receptor responses in numerous cell types, including mast cells^30^ and dendritic cells^31^, and an effective target for the treatment of autoimmune diseases and inflammation. Moreover, Syk is reported to mediate upregulation of HG-induced TGF-β1 in HK-2 cells^22^. Here, we examined the possibility that Syk inhibitors exert an anti-EMT effect in concert with FM.

FM could reduce the expression of p-Syk caused by high glucose and inhibit the TGF-β1/Smad pathway downstream of Syk. These effects were consistent and synergistic with those of the Syk inhibitor, BAY61-3606. In the CDOCKER program, scores above 20 are considered significant. As shown in Fig. 4, the small molecule inhibitors fit right into the gaps, confirming a high -CDOCKER_INTERACTION_ENERGY score. In view of the finding that FM had a lower binding energy score (−75.1069 kcal/mol) than the Syk inhibitor, LASW836 (−57.4404 kcal/mol), we expect broader prospects for application of FM in the future. Using molecular docking and SPR, we showed that FM could directly bind Syk and thereby exert a major inhibitory effect on its function. Phosphorylation of Syk can be activated under high glucose conditions, leading to stimulation of the downstream TGF-β1/Smad pathway. Our experiments additionally demonstrated that FM inhibits activation of the TGF-β1/Smad pathway induced by high glucose through suppression of Syk activation, leading to an inhibitory effect on EMT in HK-2 cells. The decrease in activity may thus be caused via direct binding and inhibition by FM.

Earlier, Yang *et al*.^22^ explored the role of Syk in HG-induced TGF-β1 upregulation in HK-2 cells. Their experiments showed that Syk serves as a mediator of intracellular signal transduction for TGF-β1 upregulation and is activated by high glucose. As one of the key fibrogenic factors, TGF-β1 is a classical regulatory cytokine and an important inducer of renal tubular epithelial cell transdifferentiation^21,32,33^. TGF-β1 is reported to exert biological effects by activating downstream mediators known as Smads. Smad proteins, the main downstream effector molecules in the TGF-β1 signaling pathway, transduce the TGF-β1 signal from the cell membrane to the nucleus. In the TGF-β1/Smad pathway, Smad proteins are phosphorylated by TGF-β1 and its receptors to regulate the expression of related genes and promote EMT development. Activated Smad3 and Smad2 subsequently bind to Smad4 to form a Smad complex, which is transported to the nucleus and works with other cofactors to regulate the transcription of target genes^34–37^. The signaling cascade from Syk to TGF-β1 and Smads thus appears to be a critical pathway. The findings that the Syk inhibitor, BAY61-3606, and Syk siRNA abrogate the influence of high glucose on EMT expression support the involvement of the signal cascade from Syk to the TGF-β1/Smad axis in the anti-EMT effect.

In conclusion, we propose that a HG-induced signal transduction pathway promotes TGF-β1 expression in HK-2 cells in which Syk plays an important role. Syk activation triggers stimulation of TGF-β1/Smad activity, implicating its involvement in diabetic kidney disease. Overall, a signaling network of FM including its direct target, Syk, was established in this study based on inverse docking and bioinformatics data. Binding of FM to Syk and activation of the downstream signaling cascade may be a critical determinant of the anti-EMT effects of FM. Based on the collective findings, we propose that the renal protective effect of FM in high glucose-exposed HK-2 cells is partially mediated via the Syk /TGF-β1/Smad signaling pathway. Further *in vivo* studies are necessary to clarify the mechanisms underlying the inhibitory effects of FM on EMT.

## Materials and Methods

### Materials and reagents

FM was purchased from Extrasynthese (Z.I Lyon Nord, Genay, France; purity ≧99.0%). BAY61-3606 was purchased from Sigma Chemical Co.(St. Louis, MO, USA; purity ≥98.0%). Human proximal tubular epithelial cells (HK-2; China Center for Type Culture Collection, CCTCC) were maintained in MEM (5.5 mM D-glucose) supplemented with 10% (v/v) fetal bovine serum (FBS), 100 μg/mL streptomycin and 100 U/ml penicillin at 37 °C with 5% CO_2_. To induce EMT, HK-2 cells were cultured for 48 h in high-glucose medium (60 mM D-glucose was selected based on preliminary experiments^38^ and previously published findings^39^). High mannitol as an osmotic control exerted minimal or no effects on EMT factors and TGF-β1^39,40^. Thus, no osmotic control was required. Upon reaching 60–70% confluence, HK-2 cells were cultured in serum-free medium for 24 h prior to experimentation. All primary antibodies were obtained from Abcam (Cambridge, MA, USA).

### Determination of the effects of FM on viability and proliferation of HK-2 cells under normal and high glucose conditions

The MTS cell proliferation assay (Promega, Madison, WI, USA) was used to determine cell viability. In total, 5000 HK-2 cells were seeded on 96-well plates in complete medium for 24 h and incubated with various concentrations of FM for 24 h and 48 h in serum-free culture medium (normal conditions) or FM in 60 mM D-glucose for 48 h in serum-free culture medium (high-glucose conditions). MTS solution (20 μL) was added to each well, followed by incubation for 2 h at 37 °C in a humidified atmosphere containing 5% CO_2_. The resulting color was assayed at 490 nm using a microplate reader (Bio-Rad Laboratories, Inc., Hercules, CA, USA).

### Prediction of direct target proteins of FM

#### Reverse screening for targets of FM

Discovery Studio 2017 R2 (NeoTrident Technology Ltd., a computer platform of Immunology Teaching and Research Department of the Basic Medical College of Peking University) was applied for identifying possible protein targets of FM. The calculation process was as follows: (1) the 3D structure of FM was constructed and the structure optimized. (2) The ligand profiler was used to perform reverse target screening of small molecules based on the pharmacophore database. (3) The score of the Fit Value increased with stronger interactions between FM and the corresponding protein.

#### Network construction and analysis

To determine the key proteins contributing to the anti-EMT function of FM, a PPI network was constructed based on the results of reverse target screening. In total, 15 human proteins were identified in reverse target screening and visualized using Cytoscape 3.6.1 platform BisoGenet Version 3.0.0 (http://www.cytoscape.org), as described previously. Subsequently, GO analysis was conducted to enrich proteins of the PPI network for biological processes.

### Confirmation of the FM target

#### Molecular docking and dynamic simulation

The CDOCKER program in Discovery Studio™ 2.5 (DS; Accelrys Software Inc., San Diego, CA, USA) based on the CHARMm force field was utilized to achieve molecular docking results. The Syk protein was obtained from Protein Data Bank (PDB ID: 3vf9, http://www.rcsb.org/) and the FM molecular preparation created according to the Zink program (http://zinc15.docking.org/). Before the docking procedure, a force field was applied and binding energy minimized. Next, a ligand-based similarity search scheme was employed and the docking protocol performed as a default setting to avoid a potential reduction in docking accuracy. The complex was solvated with water in a cubic box with an explicit periodic boundary model to stimulate the environment within the cell. After creating a harmonic restraint, a standard dynamics cascade was performed, including heating, minimization, equilibration and production dynamics^41,42^.

#### Surface plasmon resonance biosensor analysis of binding between FM and Syk

*In vitro* binding affinity between Syk and FM was evaluated using Biacore T200 surface plasmon resonance (SPR, GE Healthcare). Recombinant human Syk (hSyk) protein with >95.0% purity (RPE275Hu01) was purchased from Cloud-Clone Corp. (CCC, USA). Pipelines and chip (CM5 Chip, GE, Sweden) were pretreated with running buffer (50 mM Tris-HCl, pH 7.5, 150 mM NaCl, 10 mM MaCl_2_, 0.05% Tween, and 5% dimethyl sulfoxide). For SPR analysis, Syk was immobilized on a CM5 sensor chip. Analysis was performed at a flow rate of 30 µL/min at 25 °C. Incremental FM doses (6.25, 12.5, 25, 50, 100, and 200 μM) dissolved in the running buffer were injected into the channels, and binding responses recorded for 150 s continuously as response unit (RU) values. Association (*k*a) and dissociation (*k*d) rate constants were optimized using Biacore T200 Evaluation Software v2.0 (GE Healthcare).

### Western blot analysis

HK-2 cells were extracted with RIPA Lysis buffer (Thermo Fisher Scientific, Waltham, MA, USA) containing a protease and phosphatase inhibitor cocktail to obtain protein lysates. The lysate concentration was measured with the BCA Protein Assay Kit (Thermo Fisher Scientific, USA). A 20 μg aliquot of protein from each sample was separated via 10% SDS-PAGE at 120 V and transferred onto PVDF membrane (Millipore, Billerica, MA, USA) at 100 V. After blocking with 5% (v/v) skimmed milk in Tris-buffered saline-Tween-20 (TBST) for 2 h and washing three times for 30 min in TBST, membranes were incubated with primary antibodies at 4 °C overnight against E-cadherin (1:1000, ab15148, Abcam, USA), FN (1:8000, ab2413, Abcam, USA), α-SMA (1:500, ab5694, Abcam, USA), Vimentin (1:4000, ab137321, Abcam, USA), β-actin (1:2000, ab8227, Abcam, USA), Syk (1:2000, ab155187, Abcam, USA), p-Syk (Y525 + Y526, ab58575, 1:1000, Abcam, USA) TGF-β1 (1:1000, ab92486, Abcam, USA), Smad2 (1:1000, #5339, CST, USA), p-Smad2 (Ser465/467, #3101, 1:2000, CST, USA), and Smad4 (ab40759, 1:1000, CST, USA). Probed membranes were washed three times for 30 min in TBST and incubated with alkaline phosphatase-conjugated secondary antibody (1:5000) (Bioss, Beijing, China) for 2 h at room temperature. Protein bands were visualized using the BCIP/NBT Substrate kit (Invitrogen-Gibco, Carlsbad, CA, USA) and band densities scanned and calculated using Image Lab 5.2.1 Software (Bio-Rad, Hercules, CA, USA). The histograms corresponding to the blots were normalized to β-actin.

### Transfection of syk siRNA

Small interfering RNAs (siRNAs) targeting Syk designed and synthesized by Shanghai Genechem Co., Ltd (Shanghai, China) and negative scramble control siRNA (Shanghai Genechem Co., Ltd) were used. Transfection of siRNAs into recipient cells was performed using Polybrene (50 µg/mL). After 80 h, transfection efficiencies were validated via western blot analysis. The 1# siRNA sequence was 5′-AGGCCATCATCAGTCAGAA-3′, 2# siRNA sequence was 5′-ACAAAGACAAGACAGGGAA-3′ and 3# siRNA sequence was 5′-TTAGCATGTGACTCCTGAA-3′.

### Statistical analysis

Quantitative results are expressed as means ± SD using SPSS 22 (Chicago, IL, USA). Statistical significance was determined via one-way ANOVA for multiple comparisons with application of post hoc Dunnett’s T3 test. Post hoc tests were run only when F achieved *P* < 0.05. Differences were considered significant at *P* < 0.05 and all images obtained using the GraphPad Prism 7.0 package program (GraphPad Software, San Diego, CA, USA).

## Supplementary information

Supplemental figure. 1.

## Data Availability

All data generated or analyzed during the study are included in this published article and Supplementary Information Files.
