# Supplementary material for: Flavanomarein inhibits high glucose-stimulated epithelial-mesenchymal transition in HK-2 cells via targeting spleen tyrosine kinase
Source: Sci Rep. 2020 Jan 16;10:439. doi: 10.1038/s41598-019-57360-4 (PMC6965095; doi:10.1038/s41598-019-57360-4)
Supplement: Supplementary file 1 — Supplemental figure. 1. [file 41598_2019_57360_MOESM1_ESM.pdf]

Flavanomarein inhibits high glucose-stimulated epithelial-mesenchymal transition in HK-2 cells via targeting spleen tyrosine kinase

Nan-nan Zhang[1,2], Jin-sen Kang[2], Shuai-Shuai Liu[3], Si-Meng Gu[3], Zhi-peng Song[1,2], Feng-xiang Li[2], Li-feng Wang[4], Lan Yao[5], Tian Li[6], Lin-lin Li[2], Ye Wang[2], Xue-jun Li[3]*, Xin-min Mao[1,5]*

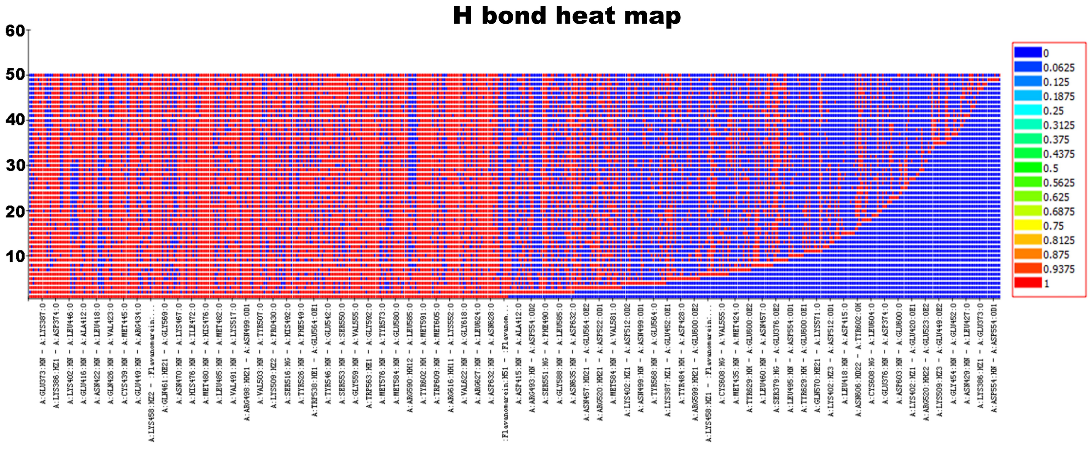

Supplemental figure. 1 Hydrogen bond heat map of the Syk -FM complex.
